# The Boston technique for acute point-of-care single-practitioner fracture stabilization of the lower extremity

**DOI:** 10.1186/s13037-019-0200-x

**Published:** 2019-05-14

**Authors:** Robert L. Parisien, Kenneth J. McAlpine

**Affiliations:** 1 Investigation Performed at Boston University School of Medicine and Medical Center, Boston, MA USA; 2 Department of Orthopaedic Surgery, Boston University School of Medicine and Medical Center, One Boston Medical Center Place, Boston, MA 02118 USA

**Keywords:** Boston technique, Orthopaedic trauma, Tibial plateau fracture, Casting technique, Acute care

## Abstract

**Background:**

Closed reduction with long leg casting is a widely practiced method of acute management of lower extremity fractures but may be cumbersome and time consuming. To our knowledge, only one method of single practitioner long leg casting has been previously reported. In this report, we describe the novel single-practitioner technique utilized at our institution for acute point-of-care temporizing management of lower extremity fractures.

**The Boston technique:**

The patient is placed supine at the edge of the hospital bed. The injured extremity is suspended from an intravenous pole in 45° of hip abduction and 30° of hip flexion. Neutral rotation is adequately maintained due to suspension through the great and second toes, without the need for patient participation. A plaster cast is applied in the usual manner and allowed to dry. Once dry, the cast is bivalved per our standard protocol to mitigate the incidence of compartment syndrome and soft-tissue complications.

**Discussion:**

The Boston technique is recommended as a single practitioner method of lower extremity fracture casting in the emergency department, trauma bay or intensive care setting. However, future studies and inclusion of additional comparable novel casting methods are required to validate our empirical findings and to further characterize the benefits and risks of casting via the Boston technique.

## Background

Acute management of lower extremity fractures in the emergency department and trauma bay is a complex and critically important step, as proper acute stabilization is crucial with respect to soft tissue integrity, fracture alignment, outcomes and patient satisfaction. Closed reduction with long leg casting is a widely accepted method of acute management of lower extremity fractures but can be cumbersome and fraught with complication. Additionally, this acute management often falls in the hands of busy orthopaedic residents and emergency medicine physicians who lack adequate labor and resources. A recently published article by The American College of Emergency Physicians highlights the difficulties in managing these critical inflection points where additional staff may be warranted [1]. With the ever increasing time-demands on individual providers and Emergency Departments, Institute for Healthcare Improvement (IHI) faculty member Kirk B. Jensen, MD, MBA stated that “the more efficient your doctors are, the less coverage you need”. The majority of the volume strain is being shouldered by house-staff as emergency department consultations, inpatient and intensive care unit (ICU) admissions drastically increase.

In close examination of emergency department trends, the Healthcare Cost and Utilization Project reported a 14.8% increase in ED visits with a total of 137.8 million visits from 2006 to 2014 [2]. However, the corresponding number of house-staff physicians has not risen at nearly the same rate. Consequently, this results in excess strain placed on individual point-of-care providers. As such, the Boston technique was developed in an effort to maximize patient safety, quality of delivered care and single-practitioner efficiency as active patient participation is not required during cast application.

Konda et al. [3] previously published their preferred casting technique for tibial shaft fractures. They report a novel technique for single practitioner long leg casting. The technique described utilizes multiple pieces of stockinette attached to both rails of a stretcher and shuttled under the leg to create a “hammock”, allowing a single practitioner the ability to cast the leg. However, this technique is only applicable if the patient is on a stretcher, which is not always the case. Furthermore, in our experience, the multiple pieces of stockinette are cumbersome, inefficient and often in the way of casting material. In addition, critically injured patients are often sent to the operating room or ICU before adequate stabilization of lower extremity fractures. We therefore present a novel technique, which can be easily utilized in all hospital settings to provide initial temporizing stabilization of lower extremity fractures.

## The Boston technique

**Fig. 1:**
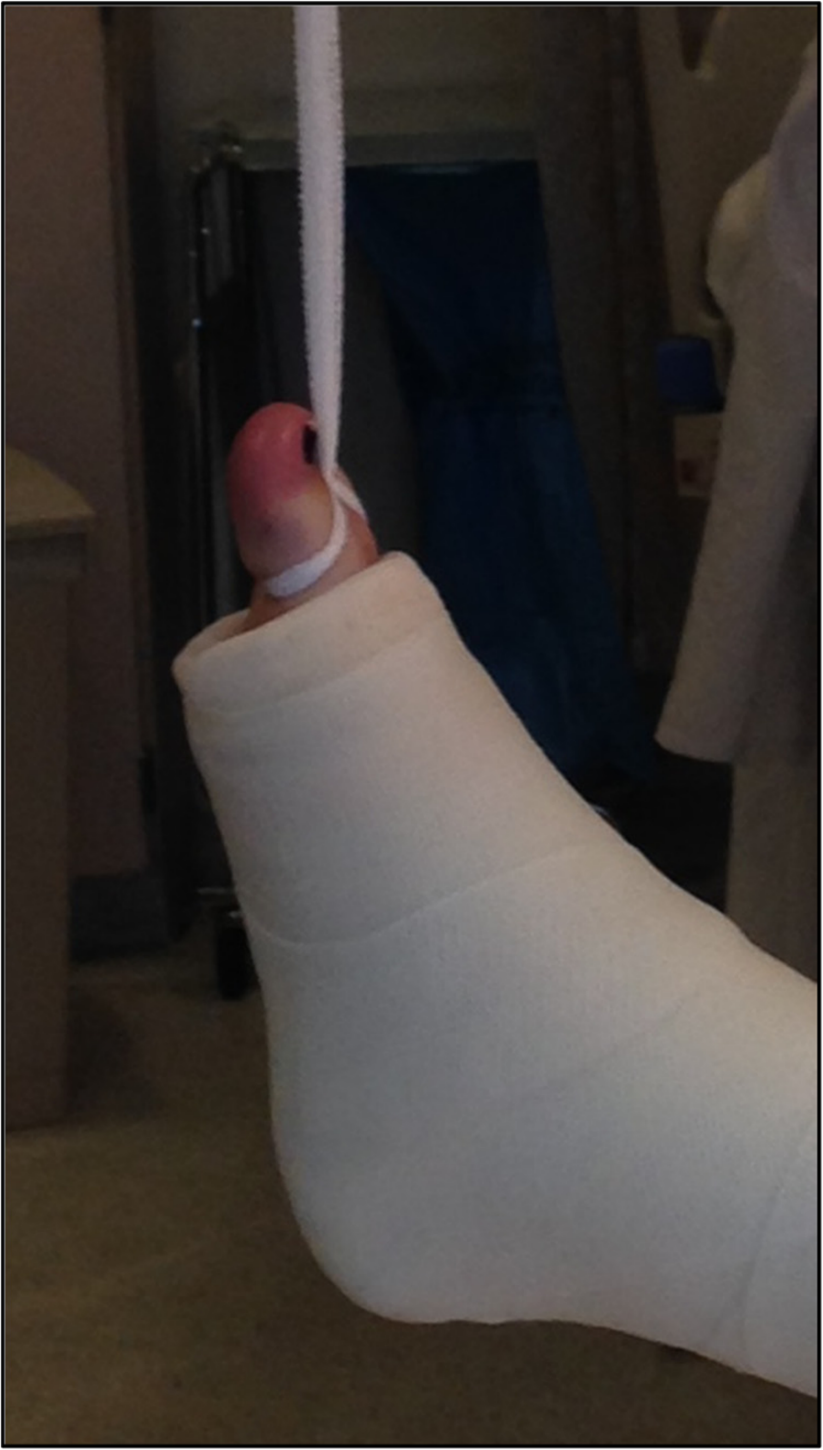
Injured extremity suspended from an intravenous pole

**Fig. 2:**
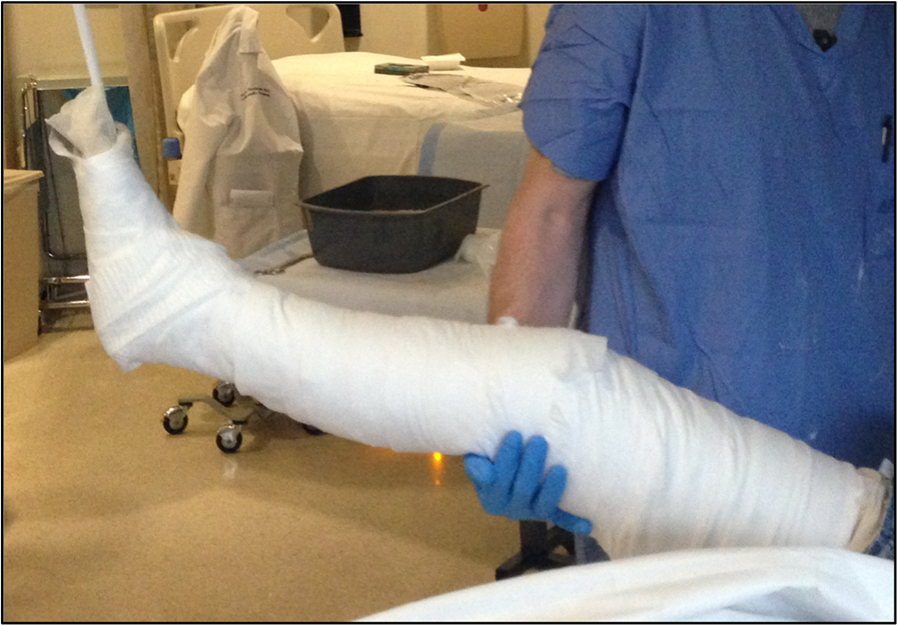
A single palm may be carefully placed in the popliteal fossa with a posterior-to-anterior directed force to adjust the desired angulation of the knee

**Fig. 3:**
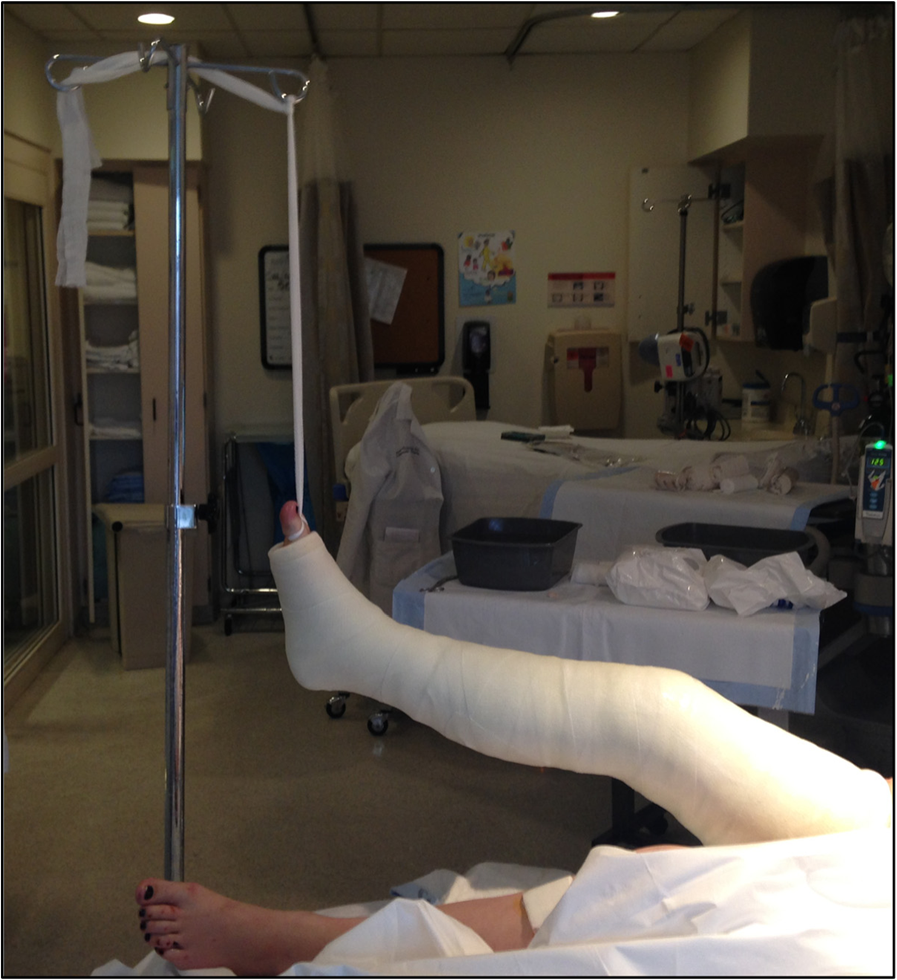
Circumferential cast material is applied by a single medical practitioner with the injured extremity suspended from the intravenous pole

**Fig. 4:**
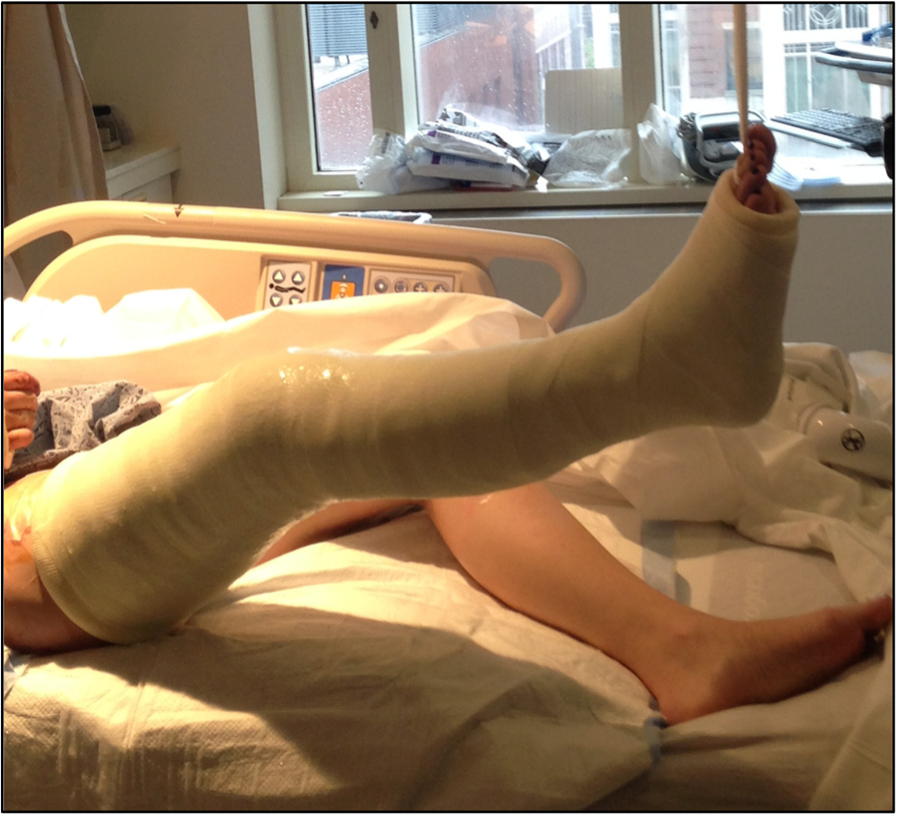
Careful suspension with appropriate positioning of the intravenous pole allows for neutral dorsiflexion of the foot and ankle

**Fig. 5:**
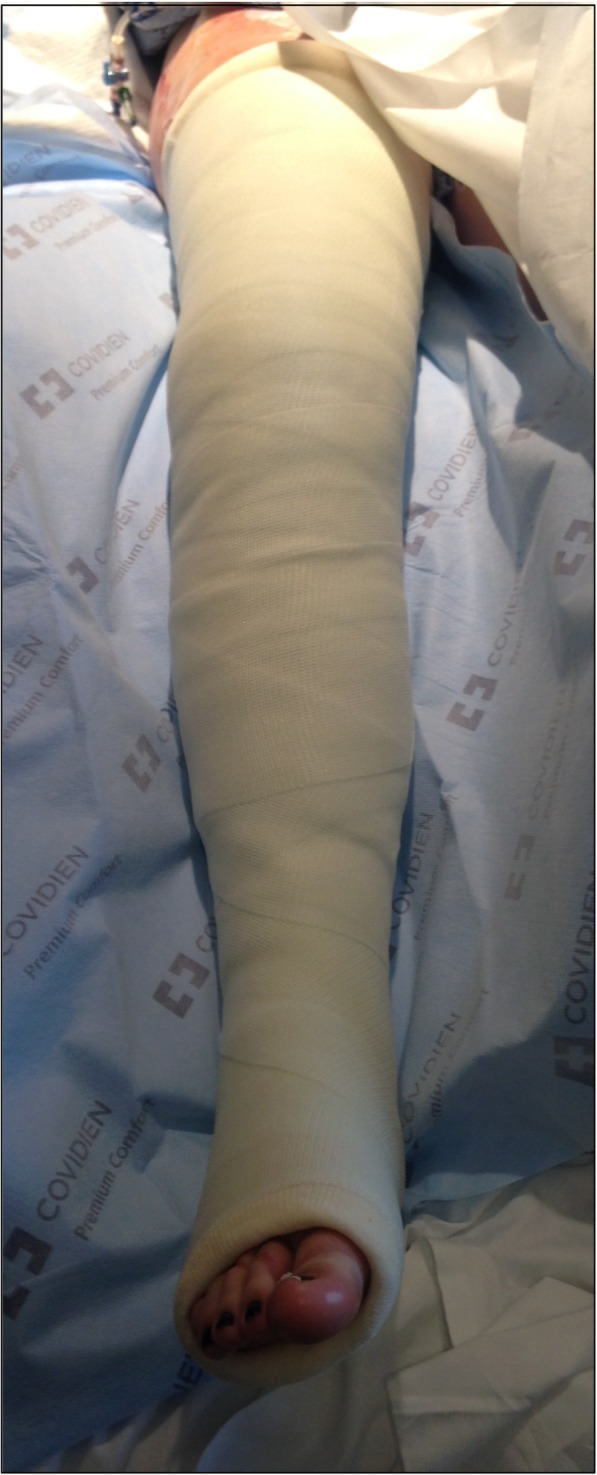
Neutral rotation is adequately maintained throughout the casting session without the need for active patient participation

The Boston technique is performed by a single medical practitioner for acute point-of-care casting and stabilization of lower extremity fractures in the emergency department, trauma bay or intensive care unit. This technique does not require active patient participation. The patient is placed supine with the ipsilateral ischial tuberosity positioned at the edge of the emergency stretcher or hospital bed. Two sections of stockinette stretch fabric are positioned over the proximal thigh and distal foot in preparation for long leg casting. A Sof-Form™ Conforming Bandage (Medline Industries, Mundelein, IL), Conform™ Stretch Conforming Bandage (Medtronic/Covidien, Minneapolis, MN) or Kerlix™ Gauze Bandage (Covidien, Mansfield, MA) is then double-looped with one loop secured around the great toe and the other loop secured around the second toe to the level of the metatarsophalangeal (MTP) joint (Fig. 1). The injured extremity is suspended from an intravenous (IV) pole in 45° of hip abduction and 30° of hip flexion. A single palm may be carefully placed in the popliteal fossa periodically with a posterior-to-anterior directed force to adjust the desired angulation of the knee throughout the casting session (Fig. 2). Depending on the size of the patient and corresponding weight of the leg, weights may be placed at the base of the IV pole for added stability. Webril™ 100% Undercast Padding (Medtronic/Covidien, Minneapolis, MN) is applied circumferentially from the level of the MTP joints to the proximal thigh(Fig. 2). The appropriate amount of circumferential fiberglass or plaster cast material is then applied by a single medical practitioner with the injured extremity suspended from the IV pole (Figs. 3 and 4). Careful suspension with appropriate positioning of the IV pole allows for neutral dorsiflexion of the foot and ankle (Figs. 3 and 4). Upon completion of hardening of the plaster or fiberglass long leg cast, the suspensory bandage is released and the leg is placed back on the emergency stretcher or hospital bed. Neutral rotation is adequately maintained throughout casting due to suspension through the great and second toes, without the need for active patient participation or corrective manipulation by the medical practitioner (Fig. 5).

While the Boston technique can be implemented in any form of long leg casting, it is mostly utilized in our institution as a way to temporize patients whom have sustained high-energy lower extremity fractures as they await definitive fixation. When used for this application, we routinely bivalve the long leg casts once the cast material is dry. As part of our standard lower extremity trauma protocol, we perform clinical compartment checks on patients having sustained high-energy injuries to the tibia every two hours for the first 24 h. Anecdotally we have found that this method, versus stabilization with a knee immobilizer or hinged brace, provides increased patient comfort and is more forgiving on the surrounding soft tissues.

For high-energy lower extremity fractures treated with a long leg cast via the Boston technique, management considerations include the degree of soft tissue compromise and fracture characteristics.. Casting via the Boston technique provides stability and soft tissue protection as patients await their definitive surgical procedure. Following surgery they are placed into either a cast or brace based on the fracture pattern and method of operative fixation utilized. In cases of significant soft tissue compromise or cases in which the patient’s medical stability precludes from immediate surgical intervention, the temporizing cast placed in the emergency department, trauma bay or ICU may require replacement following a complete 24 h period of compartment checks. In cases of intubated and sedated ICU patients, the original cast remains bivalved with clinical compartment checks continued until the patient regains the ability to participate in the examination.

## Discussion

Long leg casting of lower extremity fractures is routinely performed at trauma centers and community hospitals throughout the United States, and is widely accepted as appropriate initial management of such acute traumatic injuries. With the ever-increasing focus on access-to-care, efficiency and cost-reduction in trauma and urgent-care centers, the Boston technique represents an efficient casting method requiring only a single medical practitioner and limited emergency department resources while demonstrating consistent and reproducible outcomes with regards to quality of care and patient satisfaction [4]. From our experience at one of the highest volume Level-I trauma centers in the United States, patients consistently experience minimal discomfort and adequate limb alignment through the suspensory mechanism allowing for limited manipulation of the injured extremity in the acute setting.

There are disadvantages to using The Boston Technique for acute stabilization of lower extremity fractures. When casting, the appropriate amount of cast padding must be used to prevent thermal injury due to hardening of the cast material. If the cast is immediately bivalved prior to hardening, burn injuries from the cast saw are of clinical concern. The Boston Technique is best utilized in stable fracture patterns, whereas, casting of unstable injury patterns, including comminuted and/or segmental tibial shaft fractures, may require more than one person in cast application. The Boston technique can be employed for definitive management but, at our institution, it is mainly used as a temporizing measure. As in all casting methods, it is imperative to understand the intended goals. When using the Boston technique as a single-practitioner, controlling equinus of the ankle may prove difficult. However, our technique is an acute point-of-care temporizing method of stabilization, therefore, the mild equinus has not proven problematic as no patient is definitively treated in this position. Although we have not experienced this, we still recommend minimal immobilization in mild equinus as prolonged immobilization may result in increased ankle tightness in older patients, especially in those with pre-existing osteoarthritis of the ankle joint. As we have demonstrated, this technique has been valuable in the acute-care setting at our high-volume Level-I trauma institution. It can be employed by a single medical practitioner and provides fracture stability, soft-tissue protection, patient comfort and allows for comprehensive clinical compartment pressure management.

Furthermore, The Boston technique provides a significant cost-savings as compared to brace treatment with the average materials cost of plaster casting totaling $15 as compared to $85 for a traditional brace at our institution. A $70 per-case difference presents a substantial cost-benefit to the healthcare system when accounting for the volume of lower extremity fractures presenting to trauma centers around the country.

The Boston technique allows for the efficient and effective deployment and allocation of resources in the acute management of lower extremity fractures while minimizing discomfort, maintaining adequate fracture alignment and improving overall patient satisfaction. We therefore recommend the Boston technique as the preferred single practitioner method of lower extremity fracture casting in the emergency department, trauma bay or intensive care setting for acute point-of-care management.
